# Resectable IIIA-N2 Non-Small-Cell Lung Cancer (NSCLC): In Search for the Proper Treatment

**DOI:** 10.3390/cancers12082050

**Published:** 2020-07-25

**Authors:** Debora Brascia, Giulia De Iaco, Marcella Schiavone, Teodora Panza, Francesca Signore, Alessandro Geronimo, Doroty Sampietro, Michele Montrone, Domenico Galetta, Giuseppe Marulli

**Affiliations:** 1 Thoracic Surgery Unit, Department of Organ Transplantation and Emergency, University Hospital of Bari, 70121 Bari, Italy; deborabrascia@gmail.com (D.B.); g.deiaco24@gmail.com (G.D.I.); marcella.schiavone@gmail.com (M.S.); dorapanza91@gmail.com (T.P.); f.signore1@studenti.uniba.it (F.S.); alege_93@hotmail.it (A.G.); dorotysampietro@gmail.com (D.S.); 2 Medical Thoracic Oncology Unit, IRCCS Istituto Tumori “Giovanni Paolo II”, 70121 Bari, Italy; m.montrone@oncologico.bari.it (M.M.); galetta@oncologico.bari.it (D.G.)

**Keywords:** IIIA(N2) NSCLC, locally advanced, neoadjuvant therapy, adjuvant therapy

## Abstract

Locally advanced non-small cell lung cancer accounts for one third of non-small cell lung cancer (NSCLC) at the time of initial diagnosis and presents with a wide range of clinical and pathological heterogeneity. To date, the combined multimodality approach involving both local and systemic control is the gold standard for these patients, since occult distant micrometastatic disease should always be suspected. With the rapid increase in treatment options, the need for an interdisciplinary discussion involving oncologists, surgeons, radiation oncologists and radiologists has become essential. Surgery should be recommended to patients with non-bulky, discrete, or single-level N2 involvement and be included in the multimodality treatment. Resectable stage IIIA patients have been the subject of a number of clinical trials and retrospective analysis, discussing the efficiency and survival benefits on patients treated with the available therapeutic approaches. However, most of them have some limitations due to their retrospective nature, lack of exact pretreatment staging, and the involvement of heterogeneous populations leading to the awareness that each patient should undergo a tailored therapy in light of the nature of his tumor, its extension and his performance status.

## 1. Introduction

Locally advanced (LA) non-small cell lung cancer (NSCLC) accounts for one third of NSCLC at the time of initial diagnosis and presents with a wide range of clinical and pathological heterogeneity. Typically, LA NSCLC refers to stage III disease which represents a heterogeneous group of patients according to the most recent version of the IASLC/UICC TNM staging system (8th edition) [1]. A distinction between stage IIIA and IIIB must be carried out given the different prognosis and different diagnostic and multidisciplinary treatment approach which should be discussed case by case. Stage IIIA includes small tumors (T1a-T2b) with N2 involvement, large tumors (T3-T4) with N1 involvement and T4N0. In particular, N2 disease includes border-zone situations in which radical surgery could be predicted and suggested in a multimodality setting, along with others in which radical resection cannot be achieved and other treatment options should be evaluated. Survival of patients with stage IIIA NSCLC is poor: only half of them survive at 24 months (55%) and 5-years survival rate is only of 36% according to clinical staging [1,2]. When dealing with stage IIIA(N2), occult distant micrometastatic disease should always be suspected giving the rational for a combined multimodality approach which is the gold standard for LA tumors, since the addiction of systemic treatment to radiotherapy or surgery allows a better local and systemic control. These considerations emphasize the need for discussing every case within a multidisciplinary tumor board in which oncologist, surgeon, radiation oncologist and radiologist (at least) agree on diagnostic and therapeutic approach in order to optimize the treatment options and give the patient the best chance of cure and survival.

To date, there are still some controversies regarding the role of surgery in stage IIIA(N2) NSCLC [3,4,5], whether patients considered eligible for resection should be restaged surgically to determine operability [3,4,6,7] and whether induction regimens should include radiation therapy (RT). Although no widely agreed-upon definition of resectability exists [8,9], patients with non-bulky (defined as less than 3 cm), discrete, or single-level N2 involvement may be the best candidates to undergo resection as part of a multimodality approach. Resectable stage IIIA patients have been the subject of a number of clinical trials and retrospective analysis. Most of the published studies have some limitations due to their retrospective nature, lack of exact pretreatment staging, and the involvement of heterogeneous populations.

Given the fundamental role of surgery in managing these tumors, this review aims to discuss the main controversial points regarding the treatment of resectable stage IIIA(N2) NSCLC by searching in Literature to assess the state-of-the-art of treatment, along with new evidences and advances.

## 2. Local Control: Surgery or Radiotherapy?

The use of chemotherapy or chemoradiotherapy (CR) plus surgery should be suggested in two different subsets of stage IIIA NSCLC patients. The first subset involves patients with low tumor burden of N2 disease, considered resectable at diagnosis; in these cases, the aim of systemic therapy is to optimize distant disease control, allowing better patient tolerance and compliance. The second subset includes patients with advanced local tumors initially not amenable of surgical resection, for whom definitive CR (dCR) is the standard of care. In these cases, it is notable that by resecting the residual disease, some of these patients could be rendered disease free. The central role of chemotherapy in the setting of a multimodality therapy has been well established: Radiation Therapy Oncology Group (RTOG) 8808 study compared CR vs radiation alone in patients with stage IIIA NSCLC not amenable for surgery, a significant improvement in median survival was found with CR [10]. Moreover, two phase II trials comparing chemotherapy plus surgery to surgery alone found a similar significant improvement in median survival with chemotherapy [11,12]. In the last years, clinicians’ attention has shifted to the role of surgery following neoadjuvant therapy, since no published study has established the superiority of this approach over dCR, yet and the main concern is the potential for increase surgical morbidity and mortality [13]. Definitive concurrent CR is the standard treatment, as shown in a metanalysis by Auperin et al. [14]; by comparing six randomized trials about outcomes of concomitant over sequential dCR in patients with locally advanced NSCLC, concomitant combination increases 3-years overall survival (OS) of a 5.7% and 5-years OS of a 4.5%.

One of the limits of dCR is the high rate of local recurrence ranging between 20% and 50% [15]. A study by Caglar et al. [16] in stage IIIA-IIIB patients found out that local recurrence rates for patients who received CR plus surgery or dCR were 50% vs 7%. When examining how outcomes had changed when the dose of radiation was increased, they found no difference, although some retrospective analysis [17,18] have shown increased rates of OS for higher radiation doses, even beyond 80 Gy.

These high local recurrence rates have proved to be significantly lower when surgical resection was added after neoadjuvant therapy [5,19,20]. The role of surgical resection in the management of stage IIIA patients after induction therapy is subject of intense debate. To date, only few randomized trials comparing surgery versus radiotherapy after neoadjuvant chemotherapy have been conducted [5,20,21,22], and their results have been widely discussed in two recent meta-analyses [23,24]. None of them showed any improvement in 2- and 4-years OS; moreover, when radiotherapy was added to the neoadjuvant treatment [5,21] only 3-years progression free survival (PFS) significantly increased. Interestingly, median survival and 5-years OS rates increased over the years, starting from 19.4 vs 17.4 months in the earliest published study [22] to 49.4 vs 34.6 months in the latest [21]. These results can be explained looking at the different improvements achieved over the last years both in the surgical and radiation oncology fields. As previously described, modern radiation techniques allow a more precise targeting and higher radiation dosage in conjunction with chemotherapy, thereby avoiding irradiation to the adjacent structures as lung, esophagus, heart, and spinal cord and reducing radiation-related adverse events.

On the other hand, clinicians have started performing a better patient selection for resection procedures, by implementing preoperative studies and performing pneumonectomies only in highly selected patients [25]. It is well known, indeed, that particularly right pneumonectomy is affected by high mortality and morbidity rates [5]. Moreover, performing sleeve lobectomy can potentially reduce the surgical mortality among patients who would otherwise have required a pneumonectomy [26]. Additionally, the importance of the mediastinal clearance has been elucidated: tumors responding to neoadjuvant chemotherapy achieving a complete mediastinal response, may behave more like as de novo stage I or II disease and reflect favorable systemic chemosensitivity [27,28,29]. Factors associated with a better prognosis in patients who undergo surgery are: confirmation of a complete response of the mediastinal disease (pN0), achieving a complete resection, and confirmation of a complete pathological response.

Moreover, to achieve the best outcome, it is important to respect the perfect timing between the induction therapy and surgery which should be up to 6–8 weeks from the end of the induction treatment [30].

The most recent studies about this topic are retrospective analyses [31,32,33,34]. Aggarwal et al. [31] compared surgical treatment and dCR: lobectomy conferred a survival benefit over dCR alone (39 vs 22 months, *p* = 0.038) while pneumonectomy did not (28 vs 22 months, *p* = 0.534). Darling et al. [32] also found significant differences in median survival when comparing induction chemoradiation (iCR) plus surgery and dCR (50.4 vs 20.4 months) and higher rates of loco-regional recurrence for dCR approach (33.7% vs 51.4%). Haque et al. [34] performed a large analysis on 28,379 patients to evaluate short-term mortality following trimodality vs bimodality approaches and found a 30-day mortality rate higher in the trimodality cohort (3.4% vs 0.8%); when analyzing data per surgical technique, only pneumonectomies experienced higher 30-day mortality rates, while the lobectomies reported similar rates to those of the bimodality group. Moreover, they also compared post-treatment mortality between patients treated with dCR and those receiving iCR plus S using a video-assisted thoracoscopic surgery (VATS) approach; no significant difference in either 30- and 90-days mortality was found.

All these results suggest a potential role of trimodality approach in the treatment of certain LA NSCLC. More randomized trials are needed to explore the technical advances achieved in the last years. Moreover, the right patient selection for one specific treatment plan is peculiar in order to guarantee the best outcome; indeed, it has been already elsewhere proposed that patients requiring pneumonectomy may proportionally benefit more from up-front surgery followed by adjuvant CR than from neoadjuvant therapy [21,34]. New clinical trials should take account also for this selection criterion.

## 3. Lymph Nodes Involvement: Do the Pattern Impact on Survival?

Different rates of survival are reported in stage IIIA (N2) NSCLC, even when the same treatment approach is used. Many studies tried to investigate the weights of all factors affecting prognosis and the different lymph node metastasis pattern significantly impacts survival. Clinical vs pathological N2, multiple vs single N2 disease, and mediastinal bulky involvement are negative prognostic factors and recurrence rates proved to correlate with the subclassification of nodal involvement [35,36,37]. Robinson et al. [38] in 2007 developed the evidence-based guidelines for N2 NSCLC, classifying N2 disease in four subgroups on the basis of their behavior: IIIA1 included incidental node metastases found on final pathological examination; IIIA2 included single-station metastasis recognized intraoperatively; IIIA3 included single or multiple station disease recognized preoperatively and IIIA4 included bulky or fixed multi-station N2 disease. Extracapsular tumor spread, multiple levels of involved lymph nodes, bulky nodes, and tumor size have negative prognostic significance [39,40]. The best therapeutic approach in N2 disease remains controversial, depending also on N2 pattern [41] and should never be discussed within a multidisciplinary tumor board.

### 3.1. Unexpected N2

Mediastinal lymph node metastases may present as a wide spectrum of diseases, from microscopically single level to bulky multilevel involvement. In the absence of distant metastases, mediastinal node involvement is the most important prognostic factor in NSCLC. Consequently, preoperative assessment of lymph node status is strategical to assess the proper treatment; N2 status may require neoadjuvant therapy or exclude surgery, while N1 status should guide the decision of upfront surgical resection, to be performed either with as VATS or open surgery. However, preoperative staging is still affected by high rates of false-positives or negatives due to the relatively low sensitivity and specificity of Chest Computed Tomography (CT) and 18-fluorodeoxyglucose-positron emission tomography (18-FDG-PET), and to the reduced use of preoperative invasive staging procedures such as mediastinoscopy and endobronchial ultrasound-transbronchial needle aspiration (EBUS-TBNA) [42,43,44]. Cerfolio et al. [45] reported that, when analyzing all clinical stage patients, 14% of them had unsuspected pN2 metastases. Patients with clinical stage N0 at both CT scan and PET have low possibilities (2.9–3.7%) that subsequent mediastinoscopy or EBUS-TBNA could find unsuspected pN2 disease; in contrast, patients with clinical N1 disease suspected by CT-scan or PET present a higher incidence of unsuspected pN2 after mediastinoscopy or EBUS (17.6–23.5%) [46]. The incidence of unexpected pN2 in clinical I stage NSCLC patients has been reported in several studies, ranging from 3.5 to 16%. The role of unexpected pathological positive mediastinal lymph node metastasis (cN0-pN2) has been recently widely discussed, since this subset of patients has better survival rates than those with clinically positive nodes. Different series reported OS rates in cN0-pN2 lymph nodes of approximately 25–27%, which may increase to 35–50% with adjuvant chemotherapy [47,48,49]. Cho et al. [50], conducted an analysis on a cohort of 1,821 patients, in which unsuspected pN2 disease rate was 10.8%. After 90% of all patients succeeded in receiving complete adjuvant therapy, they reported a 5-year OS for pN2 disease of 56.1%. In their retrospective study, Fiorelli et al. [51] recently compared median OS of cN2 vs cN0pN2 patients and found a significant difference of 56 vs 20 months (*p* = 0.001).

In most cases, occult N2 disease can be found in node stations 7, 4, and 5; in particular, for station 7, its proximity to major structures with high PET-sensitivity, like the heart, may lead to misrecognition of nodal involvement [52].

Given the difficulty to assess preoperatively the nodal status, current research focused on identifying possible risk factors linked to higher rates of cN0-pN. Cho et al. [53] performed a retrospective study to determine the predictive factors for node metastasis in clinical stage I patients. They found pN1-N2 metastases in 19.4% of all cases; on multivariate analysis, large tumor size and solid consistency on preoperative radiologic exams were predictors of node metastases. Moreover, Melek et al. [54] found a false negative rate of 25% for PET in mediastinal staging, which was higher for central tumors rather than peripheral ones and for adenocarcinoma rather than other histological types. Similar findings were recently reported by Gao et al. [55]; they found that occult metastases were much more frequent in central lung cancer than in peripheral ones (17.5% vs 4.4%; *p* < 0.001). Lee et al. [56] reported that a high SUV_max_ of primary tumor (>4) could be a significant predictive factor for tumor aggressive behavior, increasing the rate of lymph node metastases from 1.9 to 10.5%. Other positive predictive factors for occult pN2 disease are younger age, being non-smoker, female gender, and PET positive uptake in N1 nodes [57,58,59,60]. Since similar findings have been reported by a number of studies, it could be speculated that patients with these characteristics should be staged with more invasive techniques and should undergo a more aggressive surgical resection. In light of these shared results, Zhang et al. [61] proposed a prediction model of N2 metastases including four independent predictors (age, tumor size, central location, and adenocarcinoma) which should guide and help to select candidates for different diagnostic and therapeutic strategies.

### 3.2. Single or Multiple-Station N2

The correct therapeutic management of IIIA(N2) disease, has widely been discussed and the question of using surgical resection rather than dCRT is almost opened. The main aim of existing research is to definitely prove the impact of the extent of lymph node metastasis on survival and to standardize treatment for each subset of patients. It has been established that several factors impact survival: the characteristics of the tumor, the presence of subcarinal disease, the extent of the resection and the number of N2 stations involved. Among all, this last factor is the one with most impact on survival [62,63]. OS for patients with single vs multiple stations after surgical resection has been analyzed in a number of studies (Table 1).

Even if the population and treatments are very heterogeneous, multi-station involvement proved to have a significant negative impact on survival in almost all reports. Several questions remain regarding both the selection of patients most appropriate for surgery in this setting and the most appropriate perioperative treatment strategy. As shown in Table 1, all kinds of treatment included surgical resection. A study by Tanner et al. [82] tried to investigate the preferences of American physicians when dealing with pN2 patients. They found that almost all clinicians (92%) included surgical treatment in patient with single-station disease whereas 48% indicated surgery for multi-station N2 disease.

Inoue et al. [36] performed a study to evaluate the most suitable pattern of pN2 NSCLC for surgical treatment. They identified patients with single N2 involvement with a primary tumor located in the upper lobe as the best candidates for surgical resection, achieving 5-years survival rates of 53.5%, comparable to that of the N1s. Similar findings were reported by Matsuguma et al. [65]; they stated that patients with multiple pN2 should not undergo to surgical resection since their outcomes compared to those with single-station disease were very poor. The main concern about these results is that 64.4% of multiple-station pN2 have been initially staged as single station cN2 and they have been discovered only after surgery on pathological examination. On the other hand, Hoffman et al. [70] recently performed an analysis on 104 N2 patients and showed that when dividing patients in groups according to the Robinson classification, all patients who underwent to surgical resection had a better OS compared to those treated conservatively (39.8 vs 19.6; *p* = 0.014). Moreover, Lee et al. [68] also suggested that even the patients with single or multi-station disease could be surgical candidates in contrast of previous beliefs that these cohort of patients were more likely to be candidate to dCR. With the aim to see whether the present N categorization accurately reflects the prognosis, Asamura et al. [83] have analyzed the IASLC Lung Cancer Staging Project database suggesting a new subdivision according to the number of stations involved and the presence or not of skip metastases. In particular, pN2 has been divided into pN2 single (pN2a) and pN2 multiple (pN2b); when comparing the survival curves for pN2a and pN2b, they were statistically significant with better OS rates for pN2a. When analyzing data on the basis of the presence of skip metastasis, instead, N2 lymph nodes were divided as follows: pN2a1 single with skip (no pN1 involvement), pN2a2 single without skip (pN1 involvement as well), and pN2b. Survival rates between pN2a1 and pN2a2, as well as between pN2a2 and pN2b was statistically different and the prognosis of pN2a1 was close to that of pN1b without nodal involvement in N2 region.

Yun et al. [84] recently reported their outcomes of upfront surgery followed by adjuvant treatment for resectable N2 diseases. Following the previously described IASCL subdivision of lymph nodes, they found similar results to those of Asamura: patients with pN2a1 had excellent survival outcomes, similar to stage II patients (5YOS 58.0%) when compared to pN2a2 (5YOS 46.2%, *p* = 0.032) and pN2b (5YOS 39.0%, *p* = 0.0006); moreover, the addition of RT to postoperative chemotherapy, had positive prognostic effect for patients with pN2b (5YOS 47.0% vs 27.9%, *p* = 0.013).

## 4. The Perfect Timing: Neoadjuvant or Adjuvant Chemotherapy?

The role of chemotherapy has been well established, and a large number of trials reported a good impact on survival in the perioperative setting, both neoadjuvant or postoperative delivery.

Few retrospective studies and no phase III study have been published about a direct comparison of outcomes of induction versus adjuvant chemotherapy delivery in stage IIIA(N2) patients and the proper timing still remains an unsolved question [85,86]. Although not direct comparison, the available systematic reviews and metanalyses reported an average 5-year survival rate of 24% for patients treated with induction therapy and 36% for those treated with adjuvant therapy [87]. Lim et al. [88] published a meta-analysis to perform an indirect comparison of the impact of preoperative vs postoperative chemotherapy in patients with operable lung cancer. They included 32 trials (22 with postoperative and 10 with peri- or preoperative chemotherapy) comprising patients with stage IA-IV NSCLC. Overall results showed no differences in OS (hazard ratio (HR) 0.80 vs 0.81) and disease-free survival (DFS) (HR 0.80 vs 0.76); this result was confirmed also when considering only stage IIIA(N2) patients. A similar analysis was performed by Berghmans et al. [85]: when comparing induction versus adjuvant therapy in addiction to standard surgical procedure, they found no statistical differences in OS for stage III patients (HR 0.65 vs 0.85). They also found that fully planned neoadjuvant chemotherapy could be administered in 71 to 100% of patients, while adjuvant chemotherapy only could be in 23.5 to 85.2%. This is important and shines a light on considering the performance of the patient at the time of diagnosis to decide the better timing of chemotherapy delivery. Previous studies have shown that pulmonary function and quality of life are at their lowest levels 4 weeks after surgery, which is exactly the time when adjuvant chemotherapy should be delivered. While young and fit patients would be able to start adjuvant treatment, older patients could not, and they would better tolerate neoadjuvant treatments.

Koshy et al. [89] published one of the largest cohorts of 11,242 stage IIIA(N2) NSCLC patients who underwent five different treatment strategies from 1998 to 2004, including iCR plus lobectomy or pneumonectomy, lobectomy or pneumonectomy plus adjuvant therapy and dCR. Among patients who underwent surgical resection, 90-day mortality was higher in the adjuvant groups (2.46% and 1.97% vs 1.6% and 0% for pneumonectomies and lobectomies respectively). The 5-years survival rates were 33.5% and 20.8%, for iCR plus lobectomy or pneumonectomy, 20.3% and 13.4% for lobectomy or pneumonectomy plus adjuvant therapy, and 10.9% and for dCR, respectively. This work, however, is biased by the pretreatment selection of patients; those with better prognostic factors and improved performance status were more likely selected to undergo neoadjuvant chemoradiation followed by surgery. However, even when this analysis was adjusted for comorbidities, the relationship between treatment and survival remained unaltered.

Boffa et al. [90] published the results of a retrospective analysis on 2,005 clinical stage III NSCLC patients who underwent surgical resection plus adjuvant or neoadjuvant treatment. A simple unadjusted survival analysis showed that preoperative and postoperative chemotherapy did not significantly impact on 5-years OS (47% vs 42%, *p* = 0.15), while preoperative CR was associated with better survival than postoperative CR (44% vs 32%, *p* < 0.001). These results may be affected by some biases which have to be considered; this analysis in fact, included clinical stage III patients, but only 32% of treated patients were confirmed to have pathologic N2 disease, leading to the presence of significant overstaging both in the preoperative and postoperative cohorts. Moreover, to adjust for potential confounders, a multivariate analysis failed to demonstrate a survival difference between preoperative and postoperative chemotherapy, with or without radiation.

## 5. Induction Therapy: Should Radiotherapy Be Included?

The majority of randomized controlled trials and meta-analyses have proven that neoadjuvant or adjuvant therapy improves survival compared with surgery alone [11,12,91,92,93,94]. Induction therapy has several advantages: it allows pathological downstaging (which is the best prognostic factor related to overall survival), it enables better local control facilitating radical surgery by decreasing the tumor volume, it may eradicate clinically undetected micrometastatic disease and it is better tolerated compared to adjuvant chemotherapy with higher full dose and cycles administration [6,95,96,97]. The main limit is the risk of disease progression during treatment losing the possibility of surgery. Although the role of induction chemotherapy (iC) is well established, the use of induction chemoradiation (iCR) remains controversial, since it allows better locoregional control rates and nodal downstaging, but not always leading to subsequent survival benefits with 5-year survival rates ranging from 21 to 41% [89,98,99,100,101,102,103].

Moreover, the most effective timing of combining the three therapeutic modalities remains unclear and one main concern is the potential toxicity, often related to higher rates of surgical complications [104].

In the 1990s, many phase II studies have been performed comparing iC and iCR outcomes of patients with stage III NSCLC, demonstrating higher pathologic complete response (pCR) rates after iCR compared to iC (26–79% vs 17–53%), even if it did not translate in overall survival (OS) benefit (27–40% vs 23–56%) [105]. In the last 20 years, only few randomized trials have been performed comparing outcomes after neoadjuvant chemotherapy or chemoradiotherapy in stage IIIA(N2) NSCLC, all with same limitations including the small sample sizes and the difficulty to enroll only stage IIIA(N2) patients (Table 2).

Interestingly, when comparing response rates, according to the WHO and Response Evaluation Criteria in Solid Tumors (RECIST) criteria [112,113], the difference was significant, in favor of the iCR in almost all cases. Moreover, like previous published findings, these recent studies confirm the trend for iCR to increase resectability rates and to decrease recurrence rate, even if these differences have not proven to be statistically significant. Despite that, none of the analyzed studies showed a statistically significant difference in OS and progression free survival (PFS) when comparing the two induction approaches. Krantz et al. [111] found that RT was associated with more than double of mortality rate at 30 and 90 days, confirming the high toxicity and proinflammatory effect of radiotherapy. Indeed, it can lead to radiation-induced pneumonitis and pulmonary fibrosis, which may delay surgical resection or interfere with the administration of preoperative chemotherapy [114]. Postoperative survival and OS could also be affected by several variables, including the type of surgery performed. In the series published by Albain et al. [115], in fact, mortality rates after iCR were 26% vs 1% when either pneumonectomy or lobectomy were performed. Thomas et al. [101] reported a postoperative mortality rate of 14% vs 6% after pneumonectomy following iCR or iC alone. In the analyzed series of patients (Table 1), perioperative mortality ranged from 0% to 6%; comparing these data to those from previous published studies (ranging from 4% to 9%), they are significantly lower maybe due to surgical and anesthesiological improvements and changed therapy patterns over time [116,117,118,119].

Additionally, radiation dose and modality matter. A study by Seder et al. [118] showed no difference in complications when patients received either 44 Gy or 60 Gy in the neoadjuvant setting. This could be interesting considering that response rate can also be related to the radiation dose; better response rates, in fact, they are typically related to higher radiation doses (>45 Gy) and hyperfractionated accelerated RT; it seems that classical fractionated RT with a total dose of 45 Gy is suboptimal in achieving local control [119,120,121,122]. In a recent study by Sher et al. [120], pathological and surgical outcomes in patients with stage IIIA NSCLC treated with differential doses of iCR (high-dose, 55–74 Gy; low-dose, 36–44 Gy; standard-dose 45–54 Gy) were compared. They found that patients treated with standard-dose RT, experienced significantly prolonged survival in comparison with those treated with lower or higher doses (*p* = 0.0089) and they were less likely to have a prolonged hospital stay. On the other hand, patients who received high-dose RT experienced the lowest probability of residual lymph-nodes disease. Additionally, hyperfractionated-accelerated RT may shorten the interval between iCR and surgery, given the reduced risk of pulmonary fibrosis [122].

Recently, results from the NRG Oncology RTOG 0617 trial demonstrated that, when associated to preoperative chemotherapy, delivery of preoperative intensity-modulated RT (IMRT) instead of three-dimensional RT (3DCRT) may boost radiation coverage of tumors by reducing normal tissues exposition leading to reduced morbidity and mortality [123].

Park et al. [124] retrospectively analyzed data about stage IIIA patients who underwent iCR followed by surgery. When performing the multivariate analysis, they found out that downstaging to pN0-1 together with age <60 years at the time of the diagnosis were independent prognostic factors for OS. Similar findings were reported by Krantz et al. [111]; their multivariate analysis, in fact, revealed older age (>65 years), male sex, comorbidity index >2 as significant risk factors for mortality.

One of the main indications for iCR is the treatment of superior sulcus tumors, as indicated by guidelines, [125] since the control of local tumor regression is the key to achieve complete resection. It has been proved, in fact, that after iCR, these tumors register high rates of complete resection and OS [126,127].

Taking into account all these parameters, it can be stated that in highly selected patients with good performance status, young age and LA disease requiring volume reduction to achieve complete resection, combined neoadjuvant chemoradiotherapy offers better outcomes in terms of pathological downstaging, even if it offers no significant improvement in terms of overall survival when compared with bimodality treatment.

## 6. Adjuvant Therapy: Should Radiation Be Included?

Completely resected IIIA(N2) stage patients have OS rates ranging from 7 to 34% and even after adjuvant chemotherapy, locoregional recurrence can be as high as 40%, independently correlating with worse OS. Several randomized trials have extensively discussed and confirmed the benefit of postoperative chemotherapy (POCT) in the subset of IIIA(N2) stage patients and it still is considered the standard of care [128,129,130]. Adding to POCT the postoperative radiotherapy (PORT) to improve locoregional control for patients with advanced NSCLC has been the subject of debate for many years, since its effect on survival has not been yet defined. Since the late 1990s many randomized trials have been conducted to prove the efficacy of PORT, until the PORT meta-analysis Trialists Group published a meta-analysis of nine randomized trials demonstrating that, when compared to surgery alone, PORT added a 21% of risk of death [131]. Although PORT detrimental effect was higher for N0 and N1 diseases, its use for N2 disease remained discretionary and it drastically felt down being applied to less than one third of the population. However, all the studies included in the PORT meta-analysis are nowadays obsolete since old data and techniques, non-standard treatment schedules and outmoded equipment as two-dimensional RT or cobalt-based RT and imprecise dosimetry were employed [132]. The Adjuvant Navelbine International Trialist Association (ANITA) trial, the results of which were published in 2008, suggested a potential benefit in median survival in patient with stage I-IIIA NSCLC when PORT was added both to the chemotherapy (47.4 vs 23.8 months) or to the observation arm (22.7 vs 12.7 months) [128]. This study, however, has several limitations: in the ANITA trial the decision to perform PORT was not randomized and its delivery was not homogeneous in the different centers. In the Surveillance, Epidemiology, and End Results (SEER) trial, instead, no details about radiotherapy and chemotherapy delivery were given. Both studies are now obsolete because of their treatment protocols and techniques. Similar results were described by Lally et al. [133] by using data from the Surveillance, Epidemiology and End Results (SEER) database about stage III patients; they observed a significant difference in 5-years OS in favor of the PORT group (27 vs 20%, *p* = 0.008); however, the SEER database did not add any information about the delivery of POCT.

In this review we looked at published evidence about the feasibility and the potential advantages of postoperative chemo-radiotherapy (POCRT) when compared to POCT alone. Studies reporting the outcomes of completely resected pN2 patients treated with POCRT or POCT alone over the last 20 years are outlined in Table 3. The main limit of these studies is their retrospective nature. Median survival rates range from 28 to 45.6 months for POCT and from 34 to 53.1 months for POCRT. Five-years survival rates range from 22.2 to 41.0% and from 30.5 to 57.5%. DFS ranging from 9.3 to 18.8% and from 14.4 to 30.3%. When comparing POCT vs POCRT rates, most of them were significantly different, confirming the potential advantage of adding the RT to the postoperative treatment. Interestingly, almost all studies which reported local relapse or local recurrence-free survival confirmed the leading role of RT in preventing local failures.

One of the main concerns about PORT is the high rate of toxicity-related disease. As a consequence of PORT, pneumonitis may occur in 1–28% of patients, as well as alterations in pulmonary function, decrease in diffusion capacity and FEV1, cardiac diseases, and esophagitis [143]. Methodological advances, as well as the use of linear accelerators and 3D-CRT, tried to limit the excess of toxic deaths, and modern PORT seems to confer an OS advantage in the treatment of N2 disease. Kepka et al. [144] conducted a prospective study comparing patients treated with PORT delivered with 3D-CRT systems and patients who did not receive PORT; they found out that the PORT group did not result in an increase of cardiopulmonary morbidity or in decrease of quality of life. Moreover, radiation doses can make the difference in toxicity; some studies demonstrated that patient receiving standard doses of PORT (45–54 Gy) after margin-negative resection, had higher survival rates than those receiving excessive doses. This highlights the importance of the preoperative and multidisciplinary evaluation of the patient, to identify the better therapeutic window in delivering PORT (patient characteristics and comorbidities, doses, techniques, and timing).

Current research aims to identify and stratify high risk patients who could benefit from PORT. Wang et al. [145], in a recent retrospective analysis on 3377 stage IIIA(N2) patients from the SEER database, compared outcomes of those treated with PORT associated or not to POCT. In a subset analysis of patients classified by the number of positive lymph nodes (*n* < 3 and *n* > 3), they found that the use of PORT significantly improves survival in patients with >3 lymph nodes, while no benefit in survival was registered in patients with <3 positive lymph nodes. Another recent study by Yuan et al. [78] compared stage IIIA(N2) patients treated with or without PORT, demonstrating that PORT could improve OS in single N2 station involved patients (65.7% vs 54.1%, *p* = 0.04), but not the multiple ones (35.3% vs 32.7%, *p* = 0.93). It has been well known that patients with pathologic single station N2 diseases are those who could get more benefit in survival from upfront surgery followed by adjuvant therapy [137]. Moreover, Zhang et al. [146] recently tried to establish whether the type of lymph node metastasis (skip or non-skip) could be predictive for prognosis in stage IIIA NSCLC treated with POCT with or without radiation. Although not significant, a trend toward improved PFS, OS and distant recurrence free survival was found in both skip and non-skip metastasis when PORT was added even if this trend was more evident in the skip metastases group. Similar findings were reported by Zou et al. [134] who found that the absence of N1 nodal involvement (skip metastases) was a good prognostic factor for both OS and DFS.

Another recent study [147] evaluated the safety and efficacy of PORT plus POCT after pneumonectomy for 119 patients with stage IIIA(N2) NSCLC, demonstrating that PORT is feasible and safe in this subset. When comparing the PORT with the non-PORT group, the median OS and DFS significantly improved in the first subset of patients, as well as locoregional failure significantly decreased in PORT group; moreover, no treatment-related complication was registered.

Another concern is that, given the lack of a consensus on the definition of the proper extent of clinical target volume (CTV), significant heterogeneity still affects all reported studies. Generally, PORT CTV includes stump, ipsilateral hilum, the initially involved lymph nodes and subcarinal lymph nodes. On margin negative patients, PORT target volume is mainly delineated at lymph node drainage area, mostly mediastinum. Several studies have tried to investigate the locoregional patterns of recurrence after surgery to plan the optimal tailored PORT CTV; it seems, in fact, that this pattern varies depending on the location of the primary tumor [148,149,150]. As result, CTV should always include the bronchial stump, the ipsilateral hilum and positive lymph nodes; station 4 and 7 should always be included due to their high incidence of relapse. Moreover, right-sided tumors generally recur unilaterally at the ipsilateral superior mediastinal nodes while left-sided lung cancers recur more frequently in the bilateral superior mediastinal nodes [148,149,150,151].

Given the advantages linked both to PORT and POCT, their optimal sequencing after surgery has been not extensively discussed and POCT-first strategy has always been first adopted even if some evidences suggest that the correct timing may play a role in survival. Lee et al. [152] published the first retrospective study on 105 patients receiving PORT-first therapy, starting from four to six weeks after surgical resection and subsequent POCT starting three to four weeks after the completion of PORT. When comparing PORT plus POCT and POCT alone, they found higher rates of 5-years OS and DFS when PORT was added (61.3% vs 29.2%, *p* < 0.001; 49.6% vs 30.5%, *p* = 0.0049). These rates are comparable to those of retrospective reports of stage IIIA(N2) patients treated with PORT. However, Lee et al. speculated that an appropriate sequence of the postoperative therapy could maximize the therapeutic effects; delaying PORT may affect the locoregional control, while PORT-first strategy can minimize the overall delay since treatment time of PORT (5–6 weeks) is shorter than that of POCT (12–16 weeks). Sura et al. [153] recently performed a retrospective analysis to determine whether the timing between postoperative chemotherapy and radiotherapy could affect outcomes. Then, 1629 patients were divided in two groups on the basis of PORT delivery time after surgery: early time to radiation (<8 weeks) and late time to radiation (>8 weeks). After propensity score analysis, median survival time was longer in the late group (48.3 vs 38.1 months, *p* = 0.006) and when analyzing whether to add concurrent or sequential POCT, results suggested that sequential chemotherapy with a late delivery of PORT led to better survival compared with concurrent POCT and/or sequential POCT with early PORT. This could be explained by the positive role of postoperative therapy delay; it may allow, in fact, a decrease in rates of toxicity and a longer time for postoperative healing.

## 7. Induction/Adjuvant Therapy: Looking at the Target-Therapy

Adjuvant chemotherapy has indisputable advantages on IIIA(N2) NSCLC, while bringing along high rates of treatment-related toxicity which may delay or force treatment discontinuation. From the necessity to improve patients’ compliance to therapies, great attention has been given in the last years to alternative therapies with better tolerability. Overall, 10% of patients with NSCLC in the United States and 35% in Asia have tumor-associated epidermal growth factor receptors (EGFR) mutations, which are predictive of response to EGFR-tyrosine kinase inhibitors (TKIs) [154]. EGFR-TKIs as first-line treatment have proved to significantly improve OS and DFS compared to chemotherapy in LA EGFR mutation-positive NSCLC [155,156,157,158,159], particularly in patients who are never-smokers, female, or present with adenocarcinoma histology with lepidic growth pattern [160,161,162]. Checkpoint inhibitors work by affecting the interactions between the immune system and the tumor. Because curative treatments remove or ablate the macroscopic tumor, timing of immunotherapy around curative treatment may influence its efficacy.

Since 2007, many retrospective and case reports have hypothesized that neoadjuvant EGFR-TKI therapy could result in N2 downstaging and better survival rates in stage IIIA patients, although they provided short follow-up terms and no definitive answer [163,164,165,166]. The available studies, support erlotinib as the best option for neoadjuvant therapy in this subset of patients, while currently no data support the use of second- or third-generation EGFR TKIs. The trial performed by Zhong et al. [167] analyzed 24 IIIA(N2) patients assigned either to the iC or the EGFR-TKI neoadjuvant arm. They found a significant better trend in the response rate (25% vs 58.1%; *p* = 0.18) favoring erlotinib, while median OS (57.3 vs 25.5 months, *p* = 0.162) and median PFS (28.9 vs 8.6 months; *p* =  0.018) were significantly higher for the iC group. As previous studies suggested, also in this case, the most common failure pattern in the erlotinib arm was distant metastasis which could be explained by a rebound effect after discontinuing TKI therapy before surgery [168]. This may promote potential residual circulating tumor cells acceleration, thus resulting in more aggressive disease, which imply that a possible better strategy could be the addition of TKI treatment to neoadjuvant chemotherapy and eventually prolonging it in adjuvant setting [169,170].

A recent phase II trial conducted by Xiong et al. [171] evaluated efficacy of erlotinib as neoadjuvant therapy in patients with resectable IIIA(N2) EGFR mutation-positive NSCLC. Although, in the small cohort of patients analyzed they found that erlotinib improved the likelihood for surgery, associated with good resection rates and favorable tolerability without life-threatening toxicities when compared with iC. The recent EMERGING-CTONG 1103 randomized phase II trial [154] analyzed outcomes of 72 resectable stage IIIA(N2) patients undergoing either neoadjuvant erlotinib or gemcitabine plus cisplatin. Median PFS was significantly longer in the erlotinib arm vs iC (21.5 vs 11.4 months, *p* < 0.001). Response rate (54.1% vs 34.3%), complete resection (73% vs 63%), and nodal downstaging (10.8% vs 2.9%) did not differ significantly; however, a better trend towards improved outcomes was registered in the erlotinib arm.

The hypothesis to use EGFR-TKI in the adjuvant setting has been investigated in the last 20 years, even if the first results from the SWOG S0023 trial [172] and BR16 study [173] in which either unresectable stage III patients or IB-IIIA NSCLC patients received adjuvant gefitinib compared to placebo, did not show any improvement in OS or disease-free survival. Over the years, other trials [174,175] and retrospective studies [176,177] have analyzed the role of EGFR-TKIs in the adjuvant setting for early-stage NSCLC patients, and their positive results in terms of survival and clinical benefits have inspired further researches in the subset of LA NSCLC.

The prospective phase II trial conducted by Li et al. [178] has been the first examining the efficacy and safety of adding EGFR-TKI as adjuvant therapy to pemetrexed-cisplatin in IIIA(N2) NSCLC patients. When comparing adjuvant C followed by gefitinib and adjuvant C alone, they found DFS (39.8 vs 27.9 months, *p* = 0.014) and 2-years overall survival (92.4% vs 77.4%, *p* = 0.07) to be significantly longer in the gefitinib group. One of the main concerns about this therapy is the best scheduling of the EGFR-TKI relative to chemotherapy. In this study, in fact, gefitinib was delivered for 6 months immediately after chemotherapy and upon disease recurrence, eight patients received further gefitinib, achieving a good postrecurrence response rate. This may lead to review the schedule of the gefitinib, as 6 months after chemotherapy could not be enough. The recent EVAN trial [148] focused on 102 IIIA (N2) EGFR mutation-positive patients treated either with adjuvant 4 cycles of vinorelbine or 2 years of erlotinib. The results showed that 3-year disease-free survival (54.2% vs 19.8%; *p* = 0.046) and OS (51% vs 20%, *p* < 0.001) were significantly longer for the EGFR-TKI group. Moreover, when stratifying patients for their characteristics, they found that there was a difference in survival and, consequently, a higher response rate to erlotinib, when patients were non-smokers, when they had EGFR mutation type exon 19 and adenocarcinomas. Moreover, the prolonged use of the erlotinib for two years did not lead to higher grades of adverse effects and toxicity in comparison of chemotherapy.

## 8. Neoadjuvant Immune Checkpoint Inhibitors: An Area of Active Research

The last years have seen a great exploit of the immune checkpoint inhibitors (ICI) and their use could potentially revolutionize lung cancer treatment, being highly selective, safe, and well tolerated. Immunotherapy drugs targeting the programmed cell death protein-1 (PD-1), programmed cell death ligand-1 (PD-L1), or cytotoxic T-lymphocyte-associated antigen 4 (CTLA-4) block the cancer-derived inhibitory signal on effector T-cells and remove residual cancer cells. The rationale for using these therapies before surgical resection is to release neoantigens from dying tumor cells while stimulating the expansion of neoantigen-specific T-cells, producing a stronger tumor-specific CD8+ T-cell response. The activity of neoadjuvant immunotherapy activity is substantially measured on major pathological response (MPR) as defined as a proportion of cancer cells in the resected tumors and lymph nodes below 10%. Forde et al. [179], in fact, have been among the firsts to test the use of nivolumab in the neoadjuvant setting for patients with stages I, II, and IIIA NSCLC. Their protocol included the delivery of two doses of nivolumab every two weeks and the surgery to be performed approximately 4 weeks after the first dose. Of the 22 patients enrolled, 23% suffered adverse events and only in one case it was of grade 3; 80% of patients survived after the first 12 months and 45% and 15% of all tumors showed a major or complete pathological response respectively. In no case the neoadjuvant therapy led to a delay in the planned surgery. Similar findings are those described by Bott et al. [180] who analyzed results on 22 patients with stage IB-IIIA NSCLC treated with the same neoadjuvant protocol as the one described previously. The pathological analysis after surgical resection showed that the major pathological response reached 45% and 10% experienced complete pathological remission.

Preliminary data from ongoing Phase II trials confirm these results [181]. Moreover, to boost their effect, some trials have associated the ICIs with chemotherapy or to a second ICI. The experience from the ongoing NEOSTAR trial, [182] in fact, compared the efficacy of nivolumab monotherapy with nivolumab plus ipilimumab dual therapy; their results show an MPR of 17% vs 33% in favor of the dual therapy. Recently, Shu et al. [183] described a trial to test the activity of the PD-L1 inhibitor atezolizumab together with platin-based chemotherapy in the neoadjuvant setting for resectable stage IB-IIIA NSCLC. Their protocol included the delivery of atezolizumab on days 1, 8 and 15 and carboplatin on day 1 of each 21-day cycle for four cycles before performing surgery. On 30 selected patients, 77% had a stage IIIA NSCLC at presentation; 57% and 33% had a major and complete pathological response, respectively. Pathological responses were observed regardless of tumor PD-L1 expression, while histological type and genetic mutations were found to be predictive of the response. Squamous cell carcinoma proved to better response to these therapies than adenocarcinomas; moreover, patients with STK11 tumor mutations did not have significant radiographic or pathological responses.

Overall, neoadjuvant immunotherapy can induce significant pathological remissions, and it has the potential for continued anti-tumor immunity. Although these promising results prove the safety and effectiveness of neoadjuvant immunotherapy, larger studies are needed to determine its best planning in terms of duration and dose. Current studies demonstrate that choosing one or another immunotherapy drug is of paramount importance, since different drugs may lead to significant difference in the MPR rates, as well as the protocol used, the timing before surgery and the association with chemotherapy regimens. Moreover, although high PD-L1 expression seems to be linked to better responses to ICIs, also PD-L1 negative patients may respond; for this reason, assessing expression in patients can contribute only minimally to clinical decision-making about suitability for treatment. One possible explanation could be the lack of standardization in testing methods with regard to antibodies used, cutoffs/thresholds for a given antibody, and differences in scoring algorithm and test sites. Finally, no trial has yet focused specifically on IIIA-N2 stage NSCLC and no shared indication for choosing the right patients who could benefit the most of these therapies has been proposed yet.

## 9. Conclusions

Treatment for resectable stage IIIA(N2) NSCLC still remains an open challenge which is still in search for a widely consensus. Given the clinical complexity and heterogeneity of this subset of patients, each one should be opportunely stratified on the basis of the biological tumor nature; subsequently, the proper tailored therapy should be discussed within a multidisciplinary tumor board in order to give the best treatment option to every patient and achieve the best outcome.

## Figures and Tables

**Table 1:** Studies comparing overall survival (OS) in IIIA-pN2 patients with either single or multi-station metastases treated with multimodality treatment over the last 20 years. Difference of values in bold is statistically significant.

Author	Year	Country	Study Period	Type of Study	Multicenter	No. of pts	Age (Median)	Treatment Modality	No. N2 Single-Level	Single Level 5YOS (%)	No. N2 Multi-Level	Multi Level 5YOS (%)
***TRIMODALITY TREATMENT***												
**Inoue et al. [36]**	2004	Japan	1980–2002	Retrospective	No	154	62	**C + S + C/R/CR** S (86) S + C (39) S + R (11) S + CR (18) *Induction tp (22)*	75	**42.7**	79	**15.5**
**Betticher et al. [64]**	2006	Switzerland	N/A	*Prospective*	Yes	75	59	**C + S + R** C + S (42) C + S + R (33)	N/A	**43.4**	N/A	**21.8**
**Matsuguma et al. [65]**	2008	Japan	1986–2003	Retrospective	No	91	N/A	**C + S + R/CR** S (36) C + S (4) C + S + CR (4) C + S + R (5) S + C (6) S + R (28) S + CR (8)	22	N/A (*p* = 0.7776)	56	N/A (*p* = 0.7776)
**Nakagiri et al. [66]**	2011	Japan	1992–2007	Retrospective	No	121	65	**C + S + C/CR** S (63) S + C (43) S + R (8) S + CR (7) *Induction tp (13)*	N/A	45.5	N/A	38.5
**Yoshino et al. [67]**	2012	Japan	2004	Retrospective	Yes	436	65	**C/CR + S + C** S (137) C + S (84) CR + S (23) R + S (1) S + C (38) S + R/CR (113)	235	**35.8**	151	**22**
**Lee et al. [68]**	2014	South Korea	1997–2011	Retrospective	No	355	N/A	**CR + S + C/R** CR + S (60) CR + S + C (84) CR + S + R (211)	102	48.3	77	39.8
**Spaggiari et al. [69]**	2015	Italy	1998–2013	Retrospective	No	141	63	**C + S + C/R/CR** C+ S (50) C + S + C (5) C + S + R (83) C + S + CR (3)	44	**35**	59	**16**
**Hofmann et al. [70]**	2018	Germany	2009–2014	Retrospective	No	104	N/A	**C/CR + S + C** S (3) S + C (3) S + CR (19) C/R + S (34) CR (37)	39	40.3	10	24.9
***ADJUVANT THERAPY***												
**Ichinose et al. [71]**	2001	Japan	1992–1993	Retrospective	No	402	63	**S + C/R** S (178) S + C (163) S + R (61)	209	**42.5**	193	**17**
**Casali et al. [72]**	2005	Italy	1990–2002	Retrospective	No	183	64	S + C S + C (183)	127	**23.8**	56	**14.7**
**Riquet et al. [73]**	2007	France	1984–2003	Retrospective	No	586	61	**S + C/R/CR** S (99) S + C (32) S + R (338) S + CR (61)	386	**28.5**	200	**17.2**
**Dai et al. [74]**	2011	China	2003–2005	Retrospective	No	221	N/A	**S + C/R/CR** S (25) S + C (100) S + R (35) S + CR (61)	N/A	**38.8**	N/A	**22.1**
**Yoo et al. [75]**	2015	South Korea	1997–2004	Retrospective	No	206	59	**S + CR/R** S (28) S + R (59) S + CR (119)	132	**45**	74	**26**
**Tamura et al. [76]**	2016	Japan	1990–2010	Retrospective	No	182	64.4 (mean)	**S + C** S (42) S + C (140)	56	35.8	126	27.7
**Xu et al. [77]**	2017	China	2009–2012	Retrospective	No	246	59	**S + C/R/CR** S (67) S + CR (88) S + C (90) S + R (1)	160	**49.4**	86	**31.1**
**Yuan et al. [78]**	2018	China	2006–2013	Retrospective	No	576	N/A	**S + C/R/CR** S (79) S + C (376) S + R (5) S + CR (116)	308	**65.7 (PORT)** 35.3 (non-PORT)	268	**54.1 (PORT)** 32.7 (non-PORT)
***NEOADJUVANT THERAPY***												
**Yang et al. [79]**	2015	USA	1995–2012	Retrospective	No	111	63	**C/CR + S** C + S (55) CR + S (56)	N/A	37.3	N/A	42.8
***SURGERY ONLY***												
**Asamura et al. [80]**	2000	Japan	1988–1997	Retrospective	No	166	N/A	S	94	**48**	72	**22**
**Iwasaki et al. [81]**	2005	Japan	1994–2003	Retrospective	No	142	N/A	S	57	**37.2**	85	**10.2**
**Sakao et al. [35]**	2006	Japan	1996–2003	Retrospective	No	53	63	S	30		23	

5YOS: 5-year overall survival; C: chemotherapy, S: surgery; R: radiotherapy; CR: chemoradiotherapy; PORT: postoperative radiotherapy.

**Table 2:** Studies reporting the outcomes of completely resected IIIA-pN2 patients treated with neoadjuvant chemotherapy or chemoradiotherapy over the last 20 years. Difference of values in bold is statistically significant.

Author	Year	Country	Study Period	Type of Study	Multicenter	No. of pts	Treatment Modality	RR (%)	Operability (%)	Peri-Operative Mortality (%)	Median Survival (mo)	PFS	Recurrence Rate (%)	Median Follow-Up (mo)
**Pezzetta et al. [106]**	2005	Switzerland	1994–2003	Retrospective	No	82	**C (36)** (Ca + Do)	**33**	-	3	-	***p* < 0.05**	**24 (67)**	53
							**CR (46)** (Ca + Do + 44 Gy)	**67**	-	4	-		**16 (35)**	53
**Girard et al. [107]**	2009	France	2003–2007	Randomized phase II trial	Yes	46	**C (14)** (Ci + G)	**57**	-	0	24.2	15.6	9 (64.3)	31.4
							**C + CR (32)** (Ci + V + 46 Gy) (Ci + P + 46 Gy)	**87**	-	0	12.5 N/A	12.5 23.9	19 (59.4)	31.4
**Higgings et al. [105]**	2009	USA	1995–2006	Retrospective	Yes	101	**C (31)** (Ca + V)	**35**	69	5	-	-	-	38
							**CR (70)** (Ca + V + 45 Gy)	**65**	84	5	-	-	-	38
**Katakami et al. [108]**	2012	Japan	2000–2005	Randomized phase III trial	Yes	58	**C (29)** (Ca + Do)	21	89.7	0	29.9	9.7	25 (86.2)	60.7
							**CR (29)** (Ca + Do + 40 Gy)	40	86.2	0	39.6	12.4	24 (82.7)	60.8
**Pless et al. [109]**	2015	Switzerland	2001–2012	Randomized phase III trial	Yes	232	**C (115)** (Ci + Do)	**44**	82	3	26.2	-	-	52.4
							**CR (117)** (Ci + Do + 44 Gy)	**61**	85	0	37.1	-	-	52.4
**Yang et al. [79]**	2015	USA	2003–2006	Retrospective	Yes	1362	**C (528)**	**46**	-	2.4	40.8	-	-	79.2
							**CR (834)**	**58**	-	3.4	39.6	-	-	79.2
**Sher et al. [110]**	2015	USA	2003–2005	Retrospective	Yes	1076	**C (376)**	-	-	-	41.1	-	-	72.7
							**CR (700)**	-	-	-	35.4	-	-	72.7
**Krantz et al. [111]**	2018	USA	2006–2012	Retrospective	Yes	1364	**C (682)**	**57**	-	2.9	55	-	-	70
							**CR (682)**	**62.6**	-	6	45.3	-	-	70

C: chemotherapy; CR: chemoradiotherapy; mo: months; RR: Response rate; CA: Carboplatin; Do: Docetaxel; Ci: Cisplatin; G: Gemcitabine; V: Vinolrebine; P: Paclitaxel.

**Table 3:** Studies reporting the outcomes of completely resected IIIA-pN2 patients treated with postoperative chemo-radiotherapy (POCRT) or POCT alone over the last 20 years. Difference of values in bold is statistically significant.

Author	Year	Country	Study Period	Type of Study	Multicenter	No. of pts	Treatment	LR (N) or LRFS (%)	DR (n) or DRFS (%)	MS (mo)	DFS	5YOS (%)	3YOS (%)	Median Follow-Up (mo)
**Zou et al. [134]**	2010	China	1998–2005	Retrospective	Yes	183	*POCT (79)*	**33.8%**	-	-	**9.3**	**22.2**	-	72
							*POCRT (104)*	**73.2%**	-	-	**14.4**	**30.5**	-	
**Dai et al. [74]**	2011	China	2003–2005	Retrospective	No	161	*POCT (100)*	-	-	33.1	-	31.9	46.7	35.1
							*POCRT (61)*	-	-	48.3	-	38.2	63.9	
**Shen et al. [135]**	2013	China	2004–2009	Randomized controlled trial	Yes	135	*POCT (70)*	**34**	**45**	28	**18.8**	27.5	-	45
							*POCRT (70)*	**18**	**32**	40	**30.3**	37.9	-	
**Feng et al. [136]**	2015	China	2005–2012	Retrospective	No	357	*POCT (287)*	**32**	**144**	-	**16.4**	**35.1**	**51.9**	34.3
							*POCRT (70)*	**2**	**41**	-	**21.6**	**57.5**	**75.3**	
**Robinson et al. [137]**	2015	USA	2006–2010	Retrospective	Yes	4483	*POCT (2633)*	-	-	40.7	-	**34.8**	55.2	22
							*POCRT (1850)*	-	-	45.2	-	**39.3**	59.3	
**Mikell et al. [138]**	2015	USA	2004–2006	Retrospective	Yes	2115	*POCT (1197)*	-	-	**38**	-	34.7	-	-
							*POCRT (918)*	-	-	**42**	-	39.8	-	
**Herskovic et al. [139]**	2016	USA	2004–2013	Retrospective	Yes	2691	*POCT (2175)*	-	-	**44.5**	-	-	-	32.3
							*POCRT (516)*	-	-	**53.1**	-	-	-	
**Zhang *et al.* [140]**	2016	China	2008–2010	Retrospective	No	220	*POCT (177)*	32.8%	23.2%	30	-	-	39.5	-
							*POCRT (43)*	39.5%	32.6%	37	-	-	51.2	
**Drake et al. [141]**	2018	USA	2006–2012	Retrospective	Yes	2031	*POCT (1149)*	-	-	45.6	-	41.0	-	-
							*POCRT (882)*	-	-	46.8	-	43.3	-	
**Wei et al. [142]**	2019	China	2013–2016	Retrospective	No	183	*POCT (105)*	50	63	29	-	-	-	38
							*POCRT (78)*	17	40	34	-	-	-	

POCT: postoperative chemotherapy; POCRT: postoperative chemoradiotherapy; LR: local relapse; LRFS: local recurrence-free survival; DR: distant relapse; DRFS: distant recurrence-free survival; MS: median survival; DFS: disease free survival; 5YOS: 5-years overall survival; 3YOS: 3-years overall survival; mo: months.
